# Age Differences in the Longitudinal Relationship between Work-Family Conflict and Alcohol Use

**DOI:** 10.1155/2014/354767

**Published:** 2014-01-28

**Authors:** Jennifer M. Wolff, Kathleen M. Rospenda, Judith A. Richman

**Affiliations:** ^1^485 SPHPI, Department of Psychiatry, University of Illinois at Chicago, 1601 West Taylor Street, M/C 912, Chicago, IL 60607, USA; ^2^481 SPHPI, Department of Psychiatry, University of Illinois at Chicago, 1601 West Taylor Street, M/C 912, Chicago, IL 60607, USA; ^3^469 SPHPI, Department of Psychiatry, University of Illinois at Chicago, 1601 West Taylor Street, M/C 912, Chicago, IL 60607, USA

## Abstract

Research on the relationship between work-family conflict and alcohol use has generally shown small effects possibly due to failure to include important individual differences relevant to the experience of work-family conflict and alcohol use, notably age. This study examined whether the relationships between aspects of work-family conflict and alcohol use variables differed by age. Participants were 543 individuals (51.2% women) from a community sample of working adults in the greater Chicagoland area who responded to a mail survey at three time points. Results showed important differences between age groups in several predictors of alcohol use. Strain versus time-based conflict had different effects on drinking, and strain-based forms of work-family conflict were related to increased problematic alcohol use depending on age. This study indicates that individual differences, particularly age, should be systematically accounted for when studying the relationship between work-family conflict and alcohol use.

## 1. Introduction

Research examining the link between work-family conflict (WFC: when work and family roles conflict with each other) and alcohol use has generally found the strength of the relationship between these variables to be small (see [1]). This may be due to a general failure to examine individual differences (other than gender (e.g., [2])) that may moderate the relationship between WFC and alcohol use (see [3]). One individual difference—and the focus of this paper—is age, an important factor in the relationship between work-family conflict and various risk factors [4]. For example, it is possible that problematic alcohol use is more likely to co-occur with WFC during early adulthood, when an individual is beginning a career and has not yet matured out of heavier drinking patterns. The purpose of the current study was to examine the longitudinal effects of WFC on various indicators of alcohol use among individuals of different ages, while controlling for caregiving responsibilities.

Greenhaus and Beutell [5] defined WFC as “a form of interrole conflict in which the role pressures from the work and family domains are mutually incompatible in some respect” (pg. 77). Theoretical models and past literature have identified subtypes of WFC, including time-based WFC, where time pressures associated with membership in one role may make it physically impossible to comply with expectations from another role; strain-based WFC, where strain symptoms (e.g., fatigue and irritability) experienced within one role intrude into the other role; and behavior-based WFC, where specific behaviors required in one role are incompatible with behavioral expectations in the other role (although behavior-based WFC has proven difficult to operationalize) [5, 6]. Recent models of the work-family interface take a bidirectional approach that distinguishes between work interfering with family (WIF) and family interfering with work (FIW), which is important because they appear to operate independently in empirical studies. One study found that hours spent in paid work predicted WIF, whereas hours spent in family work predicted FIW [7]. More recently, FIW and WIF were found to exhibit evidence of discriminant validity via differential patterns of correlations with external correlates of  WFC. For instance, WIF correlated more strongly with job stressors and FIW correlated more strongly with nonwork stressors [8]. Meta-analytic research indicates that these subtypes of WFC tend to exhibit stronger relationships with same-domain outcomes rather than cross-domain outcomes [1, 9].

Relatively few studies have examined the effects of WFC on alcohol use, a non-domain-specific outcome. However, there is evidence that alcohol use is related to WFC [2, 10–14]. Frone and colleagues found support for a longitudinal relationship, wherein WFC was positively related to heavy alcohol use four years later [15]. Wang et al. [16] found that daily WFC predicted daily alcohol use in a sample of Chinese workers. However, there are few studies which look at FIW and WIF separately as they relate to alcohol use. One study found that WIF was related, but FIW was unrelated, to frequency of heavy drinking at the bivariate level [11]. Therefore, we examined the unique effects of time-based and strain-based WIF and FIW on various indicators of alcohol consumption and problematic drinking in order to discern potential variation in these relationships.

Age has been associated with drinking behavior, stress, coping, and WFC experiences, thus it may also affect the relationship between WFC and alcohol use. However, to date, age has not been considered explicitly in the research on WFC and alcohol use. In general, alcohol use decreases throughout adulthood [17–20], but the impact of age on the experience of WFC is more ambiguous. Task-related experience on the job likely increases with age, which may protect older workers from perceiving certain experiences as stressful. However, cognitive resources tend to decline with age, which may lead to an increased negative reaction to stress in older workers [4, 21].

Although there are many factors that contribute to younger adults' increased proclivity for alcohol use compared to older individuals, one relevant factor is that younger adults may drink more as a coping strategy. One study showed that younger men and women were more likely than their older counterparts to use drugs or alcohol to handle tension or stress associated with marriage or employment [22]. Additionally, positive alcohol expectancy (e.g., feeling less stressed due to drinking alcohol) is a stronger predictor of drinking behaviors than negative expectancy (e.g., feeling sad or depressed due to drinking alcohol) in adults under the age of 35, whereas positive and negative expectancy equivalently predicted drinking behaviors in respondents between ages 35 and 60 [23]. Thus, younger adults may be more inclined to drink to ameliorate the stress of WFC than older adults.

Regarding the relationship between age and WFC, younger age has been found to correlate with higher levels of WFC. For example, younger men reported more negative spillover between work and family [18]. Furthermore, younger employees may experience higher levels of family accommodations (family care that interferes with social activities or the relationship with one's spouse), work accommodations (accommodating work to family-care responsibilities), missed work due to childcare, and higher levels of physical and financial strain [24]. Older workers may be more likely to have seniority and be well established in their careers as well as having more time off and flexibility in their schedules [25].

Matthews et al. [4] suggest that important relationships may be masked if worker age is ignored when modeling constructs relevant to the work-family interface; yet to date age has not been explicitly addressed in the research on WFC and alcohol use. To address this gap, we examined the relationship between age, WFC, and drinking in a community sample.

Specifically, this study examined the differential effects of time- and strain-based FIW and WIF on alcohol use and problematic drinking for people in different age groups based on Levinson's life course theory [26, 27]. We hypothesize that both WIF and FIW will be most strongly positively related to drinking outcomes two years later in adults under the age of 34. We expect that adults aged 34–45 will show positive yet weaker relationships between WFC variables and drinking outcomes and that those over 45 will show the weakest relationships. Finally, we explore potential sex differences in the effects of WFC on drinking outcomes, as sex has been linked to both the likelihood of experiencing WFC [28–30] and to alcohol use [31–33].

## 2. Method

### 2.1. Participants

Data for this study are derived from a three-wave survey on the prevalence of WFC, drinking outcomes, and intervening variables in a community sample of employed adults (aged 18 and older). The wave 1 (W1) sample was identified by purchasing randomly selected phone numbers for block groups within the greater Chicagoland area and screening for eligible participants from 2006 to 2008. In the case of multiple eligible respondents in the same household, the Troldahl-Carter-Bryant method of respondent selection was used to select the respondent [34, 35]. Eligibility criteria included being aged 18 or older, employment of at least 20 hours per week at some time in the past 12 months, fluency in English or Spanish, and having unpaid caregiving responsibilities. Informed consent information was provided to potential participants in the survey packet, and consent was assumed for those who returned completed questionnaires.

Of the 2,114 eligible people who agreed to be mailed a questionnaire, 1,007 (53.3% women) returned a completed questionnaire at W1, resulting in a 47.2% response rate; 713 participated at wave 2 one year later (W2; 55.2% women), resulting in a 70.8% retention rate; 689 (52.5% women) participated at wave 3 one year after W2 (W3), resulting in a 96.6% retention rate. Participants received a $30 American Express gift card incentive to complete each questionnaire. Phone screens and mail surveys were administered in English or Spanish. Special care was taken to include men and Hispanic participants. Those who remained in the study through W3 were more likely to be older (*β* = .03, *P* < .001), but there were no differences in sex or race. Participants were instructed to skip questions regarding alcohol use if they never drank alcohol. A total of 543 participants responded to the drinking questions and returned a questionnaire at all three waves (51.2% women).

Of those who participated in all three waves, the average age was 43.0 years old (SD = 9.9) at W1 and the ethnic breakdown was 15.7% Latino/a, 34.8% African American, 43.7% White, and 4.9% “other” race/ethnicity (.9% missing). A majority of participants (71.1%) indicated holding a full-time work position at some time in the past 12 months at W3, 53.5% reported a household income of $50,000 or more, 46.4% had at least a bachelor's degree, and 70.2% were married or in a committed relationship. The majority of participants were caring for children under age 18 (75.7%), while a substantial percentage cared for children over age 18 (15.4%), a spouse/partner (24.1%), or parents (21.5%). Some participants also cared for siblings, aunts or uncles, and grandparents (less than 6% each). Compared to Chicago 2007–2011 population data on employed individuals [36], which was the closest comparison year range, our sample had a significantly higher percentage of individuals working in professional occupations (e.g., business, sciences, and law) and protective service. Our sample had significantly fewer individuals working in blue-collar and service occupations (e.g., food preparation, cleaning, and maintenance), sales, and transportation occupations. The racial/ethnic makeup of our sample was similar to the City of Chicago data, despite the fact that wave 1 nonresponders (those who agreed by phone to participate in the study but did not return a questionnaire at W1) were more likely to be Latino or Black (*χ* ^2^(3) = 154.08, *P* < .001) compared to those who returned a completed questionnaire. Wave 1 nonresponders were also more likely to be males (*χ* ^2^(1) = 19.11, *P* < .001), and comparison with City of Chicago data also indicates that women were overrepresented in our data. It should be noted that although comparisons made with City of Chicago data are from samples within the same city our sample had specific inclusion requirements (e.g., respondents had to be working 20+ hours per week at some time in the past year and had to be providing unpaid care for someone in addition to working), so the extent to which these analyses describe actual selection or response biases is uncertain. Unfortunately, there is no specific regional or national data on the characteristics of employed caregivers.

### 2.2. Measures

#### 2.2.1. Work-Family Conflict

Participants completed the *Work-Family Conflict Scale *[37], a 22-item measure which assesses four dimensions of WFC: strain-based and time-based WIF and FIW. The four subscales were strain-based WIF (6 items; *α* = .83, range of interitem correlations = .27–.65, *P*'s < .001, e.g., “After work, I have little energy left for things I need to do at home”), time-based WIF (5 items; *α* = .89, range of interitem correlations = .45–.83, *P*'s < .001, e.g., “Job demands keep me from spending the amount of time I would like with my family”), strain-based FIW (6 items; *α* = .89, range of interitem correlations = .45–.69, *P*'s < .001, e.g., “Things going on in my family life make it hard for me to concentrate at work”), and time-based FIW (5 items; *α* = .82, range of interitem correlations = .32–.62, *P*'s < .001, e.g., “I would put in a longer workday if I had fewer family demands”). All items were rated on a 4-point Likert scale ranging from 1 (*never*) to 4 (*always*), and each scale was averaged across items. A confirmatory factor analysis was conducted with items loading onto their respective subscales. The model showed acceptable fit according to cutoffs reported in Hu and Bentler [38]; *χ* ^2^ = 1090.96, *P* < .001, CFI = .94, RMSEA = .07, SRMR = .03. For the complete list of items, see [37].

#### 2.2.2. Alcohol Use

Four single-item measures were used to assess alcohol use. Two items (average number of drinks per day in the last 30 days and greatest amount of alcohol consumed in a day in the last 30 days) had response options ranging from 0 (*none*) to 7 (*more than 6*) [39]. The other two items (frequency of heavy episodic drinking and drinking to the point of intoxication in the past 12 months) had responses ranging from 0 (*never*) to 7 (*5 or more times a week*) [40]. Heavy episodic drinking was defined as having 4 or more drinks in a sitting for women and 5 or more drinks for men.

#### 2.2.3. Caregiving

Whether respondents were caring for a child under the age of six was coded dichotomously (0 = no, 1 = yes). The number of children and the number of adults cared for were continuous variables.

#### 2.2.4. Age and Other Demographics

As described below, we divided the sample into three age categories: 18–33, 34–45, and 46+ years old. Gender was scored dichotomously (1 = women, 0 = men). Due to sample size considerations, race/ethnicity was coded as White = 1 and non-White = 0. Income was an ordinal scale ranging from 0 (*less than $10,000*) to 7 (*greater than $90,000*). Also due to sample size, marital status was coded as 1 = married or in a committed relationship and 0 = other marital status. The number of hours usually worked per week was a continuous variable. See Table 1 for frequencies of the categorical variables.

### 2.3. Data Analysis

Following Levinson's life course theory, age was divided into three categories roughly corresponding to times of potentially significant life transitions. Although the theory was originally based on interviews with men, similar interviews with women in various career paths were analyzed and generally found to have similar life stages and transitionary periods. Other works have also supported the application of Levinson's theory to women [41].

According to Levinson [26, 27] the early part of adulthood, ages 17–33, constitutes the “novice phase” during which people have moved past adolescence and have begun to build a life structure. During this phase, individuals are more likely than at other ages to be starting a career with lower rank and salary and to be starting a family. The combination of these life events may breed WFC and, as younger adults are also more likely to drink and have positive expectancies of drinking, the relationship between WFC and alcohol use may be at its highest compared to older adults.

In Levinson's life course theory, ages 33–45 signify the culmination of early adulthood and the midlife transition during which people become “senior members” of their particular worlds. They may have more responsibilities including continued caregiving of children and/or elderly parents, which may also result in WFC; however, we expect that individuals at this stage may have more positive or healthy ways to cope with WFC, so the relationship between WFC and alcohol use will be lower compared to the 17–33 age group. (Age 33 was included in both of Levinson's original categories. We chose to include 33-year-olds in the younger age group due to trends in delaying entry into adult roles, such as marriage and parenthood [42].)

The final category consists of individuals aged 46 and up at which time working adults are fully in the middle adult life phase. Ideally, at this time, careers have been fully realized and children are older, needing less constant care. However, many may still be providing caregiving to older individuals [43]. Because prevalence of alcohol use at this life stage tends to be lower, it is expected that the relationship between WFC and drinking will also be lower compared with younger age groups.

Analyses were linear regressions with maximum likelihood with robust standard errors predicting each W3 alcohol use variable from the W1 WFC subscales and controlling for the corresponding alcohol use variable at W1, demographic variables, and caregiving variables. We were primarily interested in the longer term effects of WFC, which is why we present results at W3. A Poisson distribution was employed to model the dependent count variables because they were positively skewed. Each set of regressions was estimated within the three age categories to determine whether the predictors differed in their relation with alcohol use across age groups. As an exploratory analysis we also estimated each model separately for men and women. When an effect was significant, the following equation was used to determine whether the effects were significantly different across age groups and sexes: *Z* = *b*_1_ − *b*_2_/√(*SE*_1_^2^ + *SE*_2_^2^) [44, 45], where *b*_1_ and *b*_2_ refer to the regression weights of two groups being compared and SE_1_ and SE_2_ refer to their respective standard errors. Missing data was handled by using full information maximum likelihood (FIML), which utilizes all available data for each analysis [46]. FIML has been shown to reduce bias resulting from missing data better than pairwise or listwise deletion [47].

## 3. Results

In the following section, results for WFC predicting alcohol effects are reported in detail. For brevity, other significant effects are excluded in text unless they were significantly different across age groups. See Tables 2–4 for full results. For all W3 alcohol outcomes, the corresponding W1 alcohol variable fairly consistently predicted W3 alcohol use. For the average number of drinks per day in the past 30 days (Table 2), W1 strain-based FIW predicted a greater number of drinks and W1 time-based FIW predicted a fewer number of drinks per day for individuals under 34 and aged 34–45, the latter of which was significantly different from the over 45 group in both cases. Strain-based WIF also negatively predicted average number of drinks per day among those under 34, but this effect was not significantly different from the other age groups. These effects were mostly contrary to the hypothesis that WFC variables would predict drinking more strongly in the youngest age group. The effect for strain-based FIW was positive and significant for the youngest and middle age group and not for the oldest age group, which partially supported the hypothesis. Time-based WIF predicted more drinks per day for those over 45, an effect which was not significantly different from the other age groups. The effect was in the hypothesized direction but the lack of age differences was contrary to the hypothesis. The number of hours usually worked per week at W1 predicted a higher average number of drinks per day at W3 for those over 45, an effect which was significantly different from the other two age groups.

There were some important sex differences in the model predicting the average number of drinks per day. In the under 34 group, there was a negative relationship between strain-based WIF and drinks per day among women that was not present among men. In the over 45 group, there was a negative relationship between time-based FIW and drinks per day among men that was not present among women. In examining age group differences within sex, the relationship between strain-based FIW and drinks per day was positive for men under 34 and between 34 and 45 and each was significantly different from the over 45 group, supporting the hypothesis. There was a negative effect between caring for children under six years old and average drinks per day among women under 34 that was significantly different from the null effect for women aged 34–45.

For most drinks consumed in a day in the past 30 days at W3 (Table 3), W1 time-based FIW predicted fewer drinks consumed in a day for those 34–45, an effect which was significantly different from the over 45 group, contrary to the hypothesis. W1 strain-based FIW was positively related to most drinks in a day for those 34–45, although this was not significantly different from the other ages, not supporting the hypothesis. Men drank more in a day at W3 than women among those over 45, an effect which was significantly different from the 34–45 group. Within age and sex, there was a negative relationship between time-based WIF and most drinks in a day for men under 34, whereas this relationship was positive for men between 34 and 45. These effects were significantly different from each other and from the over 45 group.

For frequency of W3 heavy episodic drinking in the past 12 months (Table 4), the only significant effects for the W1 WFC variables (which were strain-based WIF and FIW predicting more heavy episodic drinking for those under 34 and time-based FIW predicting less heavy episodic drinking for those 34–45) did not differ significantly across age groups. The direction of the positive effect was in accordance with the hypothesis, but the lack of significant differences across age was contrary to the hypothesis. For individuals under 34, caring for children under age six was negatively related to heavy episodic drinking at W3, an effect that was significantly different from the 34–45 age group.

For frequency of intoxication in the past 12 months (not displayed; see note for Table 4), strain-based FIW at W1 predicted more frequent W3 intoxication among those under 34 and those 34–45. The under 34 effect was different from the over 45 group, partially supporting the hypothesis. Strain-based WIF predicted more frequent intoxication for those under 34, although this effect was not significantly different from the other age groups, also partially supporting the hypothesis. Time-based FIW was negatively related to frequency of intoxication for those under 34 and those who were 34–45. These effects were not significantly different from each other or from the over 45 group. Men under 34 and over 45 were more likely to drink to intoxication; the under 34 effect was significantly different from the 34–45 group. The number of adults cared for was negatively related to W3 intoxication for those under 34 and over 45; the under 34 effect was significantly different from the other age groups. In terms of sex within age differences, women under 34 showed a positive relationship between strain-based WIF (which was significantly different from the men's effect) and strain-based FIW and drinking to intoxication. Men over 45 exhibited a positive relationship between time-based WIF and intoxication, whereas there was no relationship for women. There was a positive relationship between time-based FIW and intoxication for women over 45 which was significantly different from the null effect for men over 45 and also significantly different from the effects for women in other age groups.

## 4. Discussion

In the present study we examined the relationship between time- and strain-based FIW and WIF and various indicators of alcohol consumption and problematic drinking across three age groups over a two-year timeframe. In addition, we controlled for other individual differences, including caregiving responsibilities and demographic variables and also examined models separately by sex. In general, W1 WFC subscales and caregiving predicted W3 alcohol variables differently across age, sex, and type of alcohol use. This study provides evidence that individual differences, particularly age, should be systematically accounted for when studying the relationship between WFC and alcohol use.

We expected to find a stronger relationship between W1 WFC and increased alcohol use at W3 in the younger age group, namely, because this group is more likely to be experiencing heightened levels of stress due to beginning a career and having increasing family responsibilities, possibly not having yet “aged out” of heavier alcohol use common among youth. Additionally, younger adults are more likely to be dating, which has been associated with increased alcohol use for young women and men whose dating partners are heavier drinkers [48, 49]. This hypothesis was correct for strain-based FIW predicting frequency of intoxication in the under 34 group versus the over 45 group in the overall sample. It is possible that those exposed to a high level of strain-based WFC during their younger years may be most at risk for developing alcohol addiction over time if healthier coping techniques, particularly for managing the impact of family responsibilities on work, are not acquired.

The middle age group, those who were 34–45, showed positive relationships between strain-based FIW and the average number of drinks per day, most drinks in a day, and drinking to intoxication (note that the effects for most drinks in a day and intoxication were not significantly different from the effects in other age groups). The average number of drinks per day, which showed a significant age difference, is arguably a less problematic alcohol outcome. Perhaps, then, as individuals in midlife transition age, WFC is associated with increased consumption in certain circumstances, yet the relative danger or riskiness of the alcohol use declines. Therefore, it is important to look at different types of alcohol outcomes and the effects of WFC over time for different age groups, as increased consumption of alcohol may ultimately lead to alcohol-related problems.

Interestingly, there was a negative relationship between time-based FIW and all four alcohol use outcomes for those in the middle age group, 34–45, as well as the youngest age group predicting the average number of drinks per day and frequency of intoxication. Not all of the effects were significantly different from the other age groups, but a pattern still emerges. Possibly, feeling that family responsibilities impose on time needed to complete work tasks is related to less drinking because individuals feel that they have to spend their spare time working, whereas those who feel strained due to family interference with work may use alcohol to self-medicate feelings of distress and exhaustion. Or, perhaps those whose family responsibilities are so overwhelming that they interfere with the ability to get their jobs done simply do not have time or do not have the opportunity to consume alcohol. This is in line with previous research indicating that the number of social roles a person has is related to less drinking [50]. These findings suggest that it is important to consider both direction (FIW versus WIF) and type of WFC (time- or strain-based) when looking at relations with alcohol use.

Two caregiving variables demonstrated significant negative effects on alcohol use in the under 34 age group: caring for children under age 6 predicting less heavy episodic drinking and caring for adults predicting less frequent intoxication. This suggests that there may be less time and opportunity for certain caregivers to drink, or ultimately these overburdened caregivers may just be too exhausted to drink. Considering that there was a negative relation between time-based FIW and alcohol use for those in the middle age group and that this group was the most likely to be caring for children under age 6, it seems likely that adults with parenting responsibilities have other means of coping with time-based family interference with work. This group might also be more likely to expect negative consequences of drinking. Thus, alcohol expectancies would be an important variable to be included in future studies of the effects of age on WFC-alcohol use relationships. The other significant caregiving effects were rather sparse and generally not significantly different by age group. It would be valuable to test these relations with other well-being outcomes, such as stress or anxiety. Feeling pressured for time and increased caregiving responsibilities appear to protect against alcohol use, but they may contribute to other types of problems.

Although there were some sex differences, a consistent pattern was not obvious. One particularly interesting finding was that, for women under 34, there was a negative relationship between strain-based WIF and the average number of drinks per day and a positive relationship between strain-based WIF and drinking to intoxication that were each significantly different from the null effect for men. Perhaps strain due to WFC is protective only for younger women and only for more casual alcohol use, whereas it is a risk factor for more serious alcohol use for individuals of other ages.

There were some age-within-sex differences of note as well. The effect of strain-based FIW predicting average drinks per day for men was significant for the two younger age groups and different than the effect for men over 45. The effect for time-based WIF predicting most drinks in a day among men under 34 was negative and significantly different from the effects for men of other ages. Men aged 34–45 actually showed a negative relationship between time-based WIF and most drinks in a day, again suggesting that age and sex are important in the relationship between WFC and alcohol use. Men under 34 showed a positive relationship between income—and a negative relationship between number of hours worked—and both heavy episodic drinking and drinking to intoxication. Perhaps men who work longer do not have time to engage in these riskier types of alcohol use, whereas men working in higher income jobs have the opportunity both time-wise and financially to drink heavily.

### 4.1. Limitations and Future Directions

This study had several limitations. First, all data were self-reported, increasing the likelihood of common method bias. However, some studies have generally attested to the quality of self-reported alcohol consumption [51, 52]. Second, comparisons with demographic characteristics of workers in the city of Chicago suggest that our data may underrepresent the experience of men and of blue-collar and sales workers who are also caregivers. This could have resulted in a reduced range in our alcohol outcomes, and thus potentially an underestimation of the effects of WFC on drinking, since individuals in these groups have been reported to exhibit overall higher levels of alcohol consumption and binge drinking [53–55]. Also, generalizability of our results to these groups may be compromised. Future research should make special efforts to recruit and retain members of these groups. A third limitation was that the purchased sample included only land line phone numbers. Consequently, individuals who only have cell lines were not included in our sampling frame. Fourth, younger participants were more likely to drop out of the study by W3. As such, the generalizability of our results for this broad age group may be attenuated. Although overall there was a wide age range in this study, there were few participants in the earliest stages of workforce participation as well as in the ages closer to retirement to examine differences in WFC-alcohol relationships in these potentially important age groups. Additional research is needed to clarify the relationships between WFC and drinking behavior in these groups. It is also important to consider that in the past few decades the average age for getting married and having children is increasing [42]; therefore the increasing variability of family status should be considered in future research. Future longitudinal research in this area should make special efforts to recruit and retain both younger and older employed caregivers.

Finally, although the focus of this paper was on the importance of considering age, an individual factor, when examining the effects of WFC on drinking behavior, there are several social contextual variables that we did not include in the study that could affect the relationship between WFC and alcohol use. For example, workplace drinking norms or culture has been shown to affect employee drinking behavior [56, 57]. As such, individuals with high levels of WFC might be more likely to drink while at work or at social functions compared to those who work in organizations with prohibitive policies against alcohol consumption. Similarly, alcohol-positive peer drinking norms among one's friends or family members have been shown to increase the likelihood that WFC will lead to use of alcohol among employed workers [16]. Additionally, workers who arrive home from work only to face conflict with family members may be particularly likely to exhibit high levels of alcohol use and problematic drinking [58]. Thus, while individual factors and coping strategies are important, future research should consider the relative importance of individual factors within the context of social factors that can also influence drinking behavior in order to obtain a more complete picture of the factors that lead workers who experience WFC to exhibit increased alcohol use.

## 5. Conclusions

Despite its limitations, the present study adds to the research in this area by demonstrating the importance of considering age in the relationship between WFC and alcohol use. Additionally, this study showed that different directions and types of WFC have distinct effects on alcohol use over time and need to be considered separately in future studies on this topic. Finally, our results suggest that the type of caregiving a person is engaged in also may either catalyze or inhibit alcohol use. Taken together, the results indicate that many factors contribute to alcohol use when in the presence of WFC and individual differences are essential for understanding this relationship.

## Figures and Tables

**Table 1:** Prevalence of categorical caregiving and demographics by age group, drinkers only.

Age group	Under 34	34–45	Over 45
Race	White = 37 (7.0%)	White = 129 (24.3%)	White = 106 (20.0%)
	Other = 58 (11.0%)	Other = 103 (19.4%)	Other = 97 (18.3%)
Marital status	Married = 74 (14.0%)	Married = 185 (35.0%)	Married = 128 (24.2%)
	Other = 19 (3.6%)	Other = 47 (8.9%)	Other = 76 (14.3%)
Children under 6 cared for	60 (11%)	105 (19.3%)	25 (4.7%)
Income	<$10,000–$30,000 = 34 (6.6%)	<$10,000–$30,000 = 28 (5.5%)	<$10,000–$30,000 = 30 (5.8%)
	$30,001–$70,000 = 42 (8.2%)	$30,001–$70,000 = 77 (15.0%)	$30,001–$70,000 = 108 (21.0%)
	$70,000 or more = 27 (5.3%)	$70,000 or more = 66 (12.9%)	$70,000 or more = 101 (19.7%)

Note: numbers across columns for race, marital status, and income do not sum to 543 due to missing data. Income is presented as a categorical variable for descriptive purposes but was modeled as a continuous variable in analyses.

**Table 2:** Unstandardized regression results predicting average number of drinks per day (W3).

Sex	Both sexes			Men			Women		
Age	Under 34 (*n* = 73)	34–45 (*n* = 149)	Over 45 (*n* = 117)	Under 34 (*n* = 28)	34–45 (*n* = 83)	Over 45 (*n* = 70)	Under 34 (*n* = 45)	34–45 (*n* = 66)	Over 45 (*n* = 47)

W1 variable	*β* (SE)	*β* (SE)	*β* (SE)	*β* (SE)	*β* (SE)	*β* (SE)	*β* (SE)	*β* (SE)	*β* (SE)

Work-family time	.06 (.09)	.11 (.09)	.24 (.09)*	−.21 (.15)	.17 (.12)	.32 (.12)*	.06 (.14)	.03 (.14)	.07 (.11)
Work-family strain	−.22 (.11)^∗†^	−.07 (.10)	−.15 (.12)	.03 (.12)	−.07 (.10)	−.06 (.12)	−.32 (.12)*	−.05 (.21)	−.26 (.15)
Family-work time	−.29 (.14)*	−.41 (.10)^∗a^	−.09 (.10)^†b^	−.49 (.17)*	−.45 (.17)*	−.23 (.11)*	−.13 (.21)	−.30 (.12)*	.08 (.14)
Family-work strain	.39 (.10)*	.32 (.09)^∗a^	−.05 (.10)^b^	.42 (.13)^∗x^	.35 (.15)^∗x^	−.03 (.12)^y^	.18 (.16)	.24 (.13)	−.05 (.18)
Sex	−.21 (.14)	−.03 (.15)	−.16 (.10)						
Race	−.15 (.18)	.08 (.12)	−.08 (.09)	.07 (.38)	.03 (.15)	−.07 (.13)	−.22 (.20)	.13 (.14)	−.17 (.14)
Marital status	.21 (.18)	.32 (.16)*	−.09 (.14)	.48 (.42)	.15 (.30)	−.12 (.14)	.22 (.10)	.35 (.22)	−.09 (.18)
Income	−.05 (.04)	−.07 (.04)*	−.05 (.04)	−.20 (.11)	−.06 (.05)	−.08 (.04)	−.01 (.04)	−.09 (.08)	−.04 (.05)
Hours usually worked	.00 (.00)^a^	−.01 (.00)^a^	.01 (.00)^∗b^	.01 (.01)*	−.01 (.01)*	.01 (.00)*	.00 (.00)	.00 (.01)	.01 (.01)*
Children cared for	−.04 (.07)	.01 (.04)	−.01 (.03)	−.05 (.12)	−.02 (.05)	−.01 (.04)	−.03 (.08)	.11 (.06)	−.07 (.07)
Children under 6 cared for	−.25 (16)	.02 (.09)	−.18 (.12)	−.16 (.37)	−.08 (.12)	−.19 (.12)	−.50 (.19)^∗x^	.10 (.16)^y^	.11 (.25)
Adults cared for	−.02 (.08)	−.04 (.07)	−.05 (.04)	−.25 (.13)	−.08 (.10)	.00 (.06)	−.04 (.09)	.03 (.10)	−.06 (.05)
Avg drinks per day (W1)	.15 (.04)*	.13 (.03)*	.20 (.03)*	.17 (.06)*	.14 (.04)*	.19 (.02)*	.15 (.07)*	.07 (.06)^x^	.27 (.07)^∗y^

Notes: W1: wave 1; W3: wave 3. *P < .05. ^†^A significant sex difference not taking age into account. *P* < .05. ^a,b^Significant age differences not taking sex into account. *P* < .05. ^x,y,z^Age differences within sex. *P* < .05.

**Table 3:** Unstandardized regression results predicting most drinks in a day (W3).

Sex	Both sexes			Men			Women		
Age	Under 34 (*n* = 73)	34–45 (*n* = 152)	Over 45 (*n* = 121)	Under 34 (*n* = 28)	34–45 (*n* = 86)	Over 45 (*n* = 72)	Under 34 (*n* = 45)	34–45 (*n* = 66)	Over 45 (*n* = 49)

W1 variable	*β* (SE)	*β* (SE)	*β* (SE)	*β* (SE)	*β* (SE)	*β* (SE)	*β* (SE)	*β* (SE)	*β* (SE)

Work-family time	−.12 (.08)	.08 (.07)	.10 (.08)	−.25 (.11)^∗x^	.21 (.07)^∗y^	.13 (.12)^y^	−.09 (.12)	−.06 (.12)	−.02 (.09)
Work-family strain	.09 (.07)	−.03 (.09)	.00 (.08)	.14 (.08)	−.08 (.09)	−.03 (.11)	.04 (.12)	.08 (.18)	.05 (.10)
Family-work time	−.19 (.10)	−.26 (.08)^∗a^	.02 (.08)^b^	−.25 (.09)*	−.24 (.13)	−.01 (.12)	−.09 (.17)	−.21 (.10)*	−.03 (.16)
Family-work strain	.10 (.09)	.17 (.08)*	.00 (.09)	.21 (.09)*	.13 (.11)	−.02 (.11)	.04 (.18)	.13 (.12)	.10 (.17)
Sex	−.09 (.12)	.00 (.09)^a^	−.30 (.09)^∗b^						
Race	−.06 (.12)	.12 (.08)	.09 (.10)	−.23 (.12)^∗x^	.08 (.10)^y^	.04 (.15)	.08 (.17)	.13 (.12)	.27 (.13)*
Marital status	.06 (.16)	.12 (.11)	−.06 (.09)	.30 (.15)	.02 (.17)	−.05 (.13)	.03 (.26)	.32 (.18)	−.19 (.14)
Income	.00 (.03)	.00 (.03)	−.03 (.03)	−.07 (.05)	.03 (.04)	−.04 (.04)	.00 (.04)	−.06 (.06)	.00 (.04)
Hours usually worked	.00 (.00)	.00 (.00)	.00 (.00)	.01 (.00)	−.01 (.00)	.00 (.00)	.00 (.00)	.01 (.00)	.00 (.04)
Children cared for	−.05 (.06)	−.01 (.03)	−.02 (.04)	−.15 (.07)*	−.03 (.03)	−.01 (.06)	−.01 (.09)	.02 (.06)	−.01 (.08)
Children under 6 cared for	−.13 (.11)	.11 (.07)	.03 (.12)	.07 (.11)	.06 (.08)	.01 (.14)	−.27 (.22)	.11 (.12)	.18 (.33)
Adults cared for	−.04 (.07)	−.09 (.06)	.00 (.03)	−.11 (.08)	−.20 (.08)^∗x^	.02 (.05)^y^	−.00 (.11)	.08 (.08)	−.02 (.03)
Most drinks in a day (W1)	.15 (.02)*	.13 (.02)*	.18 (.02)*	.17 (.02)*	.13 (.03)*	.17 (.03)*	.14 (.05)*	.13 (.03)*	.22 (.03)*

Notes: W1: wave 1; W3: wave 3. *P < .05. ^†^A significant sex difference not taking age into account. *P* < .05. ^a,b^Significant age differences not taking sex into account. *P* < .05. ^x,y,z^Age differences within sex. *P* < .05.

**Table 4:** Unstandardized regression results predicting frequency of drinking to intoxication (W3).

Sex	Both sexes			Men			Women		
Age	Under 34 (*n* = 77)	34–45 (*n* = 174)	Over 45 (*n* = 152)	Under 34 (*n* = 30)	34–45 (*n* = 93)	Over 45 (*n* = 83)	Under 34 (*n* = 47)	34–45 (*n* = 81)	Over 45 (*n* = 67)

W1 variable	β (SE)	β (SE)	β (SE)	β (SE)	β (SE)	β (SE)	β (SE)	β (SE)	β (SE)

Work-family time	−.28 (.16)	−.14 (.13)	.14 (.25)^†^	.48 (.34)	.13 (.19)	.37 (.19)*	−.18 (.24)	−.27 (.23)	−.35 (.25)
Work-family strain	.42 (.12)^∗†^	.07 (.18)	.03 (.20)	−.07 (.20)	−.09 (.18)	.03 (.21)	.50 (.20)*	.30 (.36)	−.14 (.41)
Family-work time	−.33 (.13)*	−.48 (.16)*	.01 (.20)^†^	−.25 (.12)	−.27 (.21)	−.01 (.24)	−.31 (.12)^∗x^	−.63 (.30)^∗x^	1.07 (.36)^∗y^
Family-work strain	.56 (.18)^∗a^	.35 (.16)*	−.06 (.23)^b^	.19 (.20)	.17 (.18)	−.28 (.26)	.62 (.23)*	.49 (.29)	−.11 (.39)
Sex	−.81 (.19)^∗a^	−.10 (.20)^b^	−.46 (.20)*						
Race	−.40 (.21)^†^	−.16 (.17)	.38 (.25)^†^	.79 (.50)	−.24 (.20)	.23 (.33)	−.63 (.31)^∗x^	−.16 (.31)^x^	.97 (.41)^∗y^
Marital status	−.08 (.32)	.06 (.24)	−.23 (.27)^†^	−.26 (.50)	−.44 (.41)	.05 (.31)	−.04 (.36)^x^	.65 (.38)^x^	−1.22 (.43)^∗y^
Income	.11 (.06)^†^	.09 (.05)	.00 (.09)	.52 (.18)^∗x^	.17 (.08)*	−.08 (.11)^y^	.08 (.07)	−.03 (.10)	.01 (.15)
Hours usually worked	−.01 (.01)^†^	−.01 (.01)	.01 (.01)	−.09 (.02)^∗x^	−.01 (.01)^y^	.01 (.01)^y^	.00 (.01)	.00 (.01)	.02 (.01)*
Children cared for	−.08 (.11)	.06 (.09)	.07 (.09)	.24 (.17)	.15 (.11)	.03 (.10)	−.22 (.19)	−.14 (.12)	−.12 (.19)
Children under 6 cared for	−.22 (.31)	.35 (.18)*	−.11 (.28)	−.63 (.62)	.17 (.18)	−.12 (.29)	−.29 (.39)	.70 (.33)*	1.15 (.84)
Adults cared for	−.28 (.14)^∗a^	.15 (.13)^b^	−.36 (.12)^∗†b^	.12 (.22)	.02 (.18)	−.25 (.15)	−.43 (.19)^∗x^	.46 (.22)^∗y^	−.80 (.20)^∗z^
Freq. of intoxication (W1)	.41 (.05)^∗a^	.39 (.04)*	.54 (.06)^∗†b^	.62 (.12)^∗x^	.37 (.04)^∗y^	.52 (.08)*	.42 (.08)^∗x^	.50 (.17)^∗x^	.93 (.13)^∗y^

Notes: W1: wave 1; W3: wave 3. *P < .05. ^†^A significant sex difference not taking age into account. *P* < .05. ^a,b^Significant age differences not taking sex into account. *P* < .05. ^x,y,z^Age differences within sex. *P* < .05.

Results of heavy episodic drinking (defined as 4 or more drinks for women and 5 or more drinks for men in a sitting) as the dependent variable are not shown. Results were the same as the present table or nonsignificant except for the following: there was a negative relation between caring for children under the age of 6 and heavy episodic drinking for those under 34, both sexes; there was a positive relation between hours worked and heavy episodic drinking for men over 45; there was a negative relation between time-based WIF and a positive relation between number of children cared for and heavy episodic drinking for women over 45.
